# Correction to Heating of Metallic Orthodontic Devices During Anti‐Aging Treatment With Vacuum and Electromagnetic Fields: in Vitro Study

**DOI:** 10.1111/srt.70311

**Published:** 2025-12-21

A. Scarano, D. Amuso, R. Amore, S. A. Gehrke, and S. R. Tari, “Heating of Metallic Orthodontic Devices During Anti‐Aging Treatment With Vacuum and Electromagnetic Fields: In Vitro Study,” *Skin Research and Technology* 30, no. 4 (2024): e13687, https://doi.org/10.1111/srt.13687.

The published article contains overstated conclusions and incomplete/inaccurate statements. The authors wish to correct the following sections of the published article to ensure accuracy and clarity:

**Abstract**

**Original**:

“In conclusion, anti‐aging therapy with V‐EMF causes a thermal increase on orthodontic brackets that is not harmful to pulp health.”

**Corrected**:

“In conclusion, anti‐aging therapy with V‐EMF causes a thermal increase on orthodontic brackets. Within the limitations of the in vitro model used, the observed temperature rise remained within safety thresholds, suggesting that V‐EMF therapy may be considered safe for orthodontic appliances.”

**Materials and Methods**

**Addendum**:

“Porcine muscle tissue was used to simulate the soft tissues of the cheek, which are predominantly composed of muscle. This choice aimed to reproduce, in vitro, the thermal conditions occurring in the perioral region during anti‐aging treatments.”

**Conclusion**

**Original**:

“Therefore, V‐EMF therapy can be safely performed in patients with fixed orthodontic appliances.”

**Corrected**:

“Within the limitations of this in vitro study, V‐EMF therapy did not produce temperature increases beyond established safety thresholds. Further in vivo studies are necessary to confirm the safety of this therapy in patients with fixed orthodontic appliances.”

**Ethics Statement**

**Original**:

“The investigation has been conducted in accordance with the Declaration of Helsinki and Good Clinical Practice Guidelines. The patients submitted the informed consent for the treatment and the anonymous data publishing.”

**Corrected**:

“The study was conducted entirely in vitro. The porcine muscle tissue used was obtained from commercially available sources intended for food consumption. No animals were sacrificed specifically for this research, and ethical approval was not required under current institutional and national guidelines.”

The authors apologize for these issues and for the inconvenience they may have caused.

